# The *Physiotherapy eSkills Training Online* resource improves performance of practical skills: a controlled trial

**DOI:** 10.1186/1472-6920-12-119

**Published:** 2012-11-26

**Authors:** Elisabeth Preston, Louise Ada, Catherine M Dean, Rosalyn Stanton, Gordon Waddington, Colleen Canning

**Affiliations:** 1 Discipline of Physiotherapy, The University of Sydney, Sydney, Australia; 2 Discipline of Physiotherapy, Macquarie University, Macquarie, Australia; 3 Discipline of Physiotherapy, The University of Canberra, Canberra, Australia

**Keywords:** E-learning, Physiotherapy students, Stroke, Practical skills

## Abstract

**Background:**

E-learning is a common and popular mode of educational delivery, but little is known about its effectiveness in teaching practical skills. The aim of this study was to determine whether the *Physiotherapy eSkills Training Online* resource in addition to usual teaching improved the performance of practical skills in physiotherapy students.

**Method:**

This study was a non-randomised controlled trial. The participants were graduate entry physiotherapy students enrolled in consecutive semesters of a neurological physiotherapy unit of study. The experimental group received the *Physiotherapy eSkills Training Online* resource as well as usual teaching. The *Physiotherapy eSkills Training Online* resource is an online resource incorporating (i) video-clips of patient-therapist simulations; (ii) supportive text describing the aim, rationale, equipment, key points, common errors and methods of progression; and (iii) a downloadable PDF document incorporating the online text information and a still image of the video-clip for each practical skill. The control group received usual teaching only. The primary outcomes were the overall performance of practical skills as well as their individual components, measured using a practical examination.

**Results:**

The implementation of the *Physiotherapy eSkills Training Online* resource resulted in an increase of 1.6 out of 25 (95% CI −0.1 to 3.3) in the experimental group compared with the control group. In addition, the experimental group scored 0.5 points out of 4 (95% CI 0 to 1.1) higher than the control group for ‘effectiveness of the practical skill’ and 0.6 points out of 4 (95% CI 0.1 to 1.1) higher for ‘rationale for the practical skill’.

**Conclusion:**

There was improvement in performance of practical skills in students who had access to the *Physiotherapy eSkills Training Online* resource in addition to usual teaching. Students considered the resource to be very useful for learning.

## Background

Teaching and learning has historically occurred in a face-to-face context. However, with the advent of the internet, e-learning has become a common mode of educational delivery. E-learning is popular with students since study can be carried out at the convenience of the learner and it also allows for revision of content. It is popular with educational institutions because it allows flexible delivery and appears to be cost effective [1]. The dramatic advances in technology in the last decade have also altered the expectation of students, such that most expect at least part of their university education to encompass some form of e-learning. Little is known, however, about its effectiveness, particularly in teaching practical skills.

Effective performance of practical skills is important across a range of health professions. Practical skills required in physiotherapy incorporate a wide range of manual techniques and exercise strategies. Learning practical skills requires practice and this can be enhanced in several ways. Observation of the skill, combined with physical practice, has been shown to promote skill learning [2]. Observation may be effective because it allows the learner to attend to subtleties of the skill [2]. Furthermore, learning practical skills may be enhanced when the learner has some control over the practice conditions, such as the length and/or the order of practice [3]. For example, learning a sporting skill was found to be better in participants who were provided with a choice of when and how often to observe a video model, compared with participants who had no choice in the timing and frequency of observation [4]. Until recently, practical skills in physiotherapy curricula have been taught based on live demonstration, followed by practice and feedback, at a time and manner determined by classroom and time constraints. This leaves students to revise the skill outside class time based on memory or on hand-written, potentially inaccurate, notes. Consequently, current teaching of practical skills may not be optimal.

**Figure 1:**
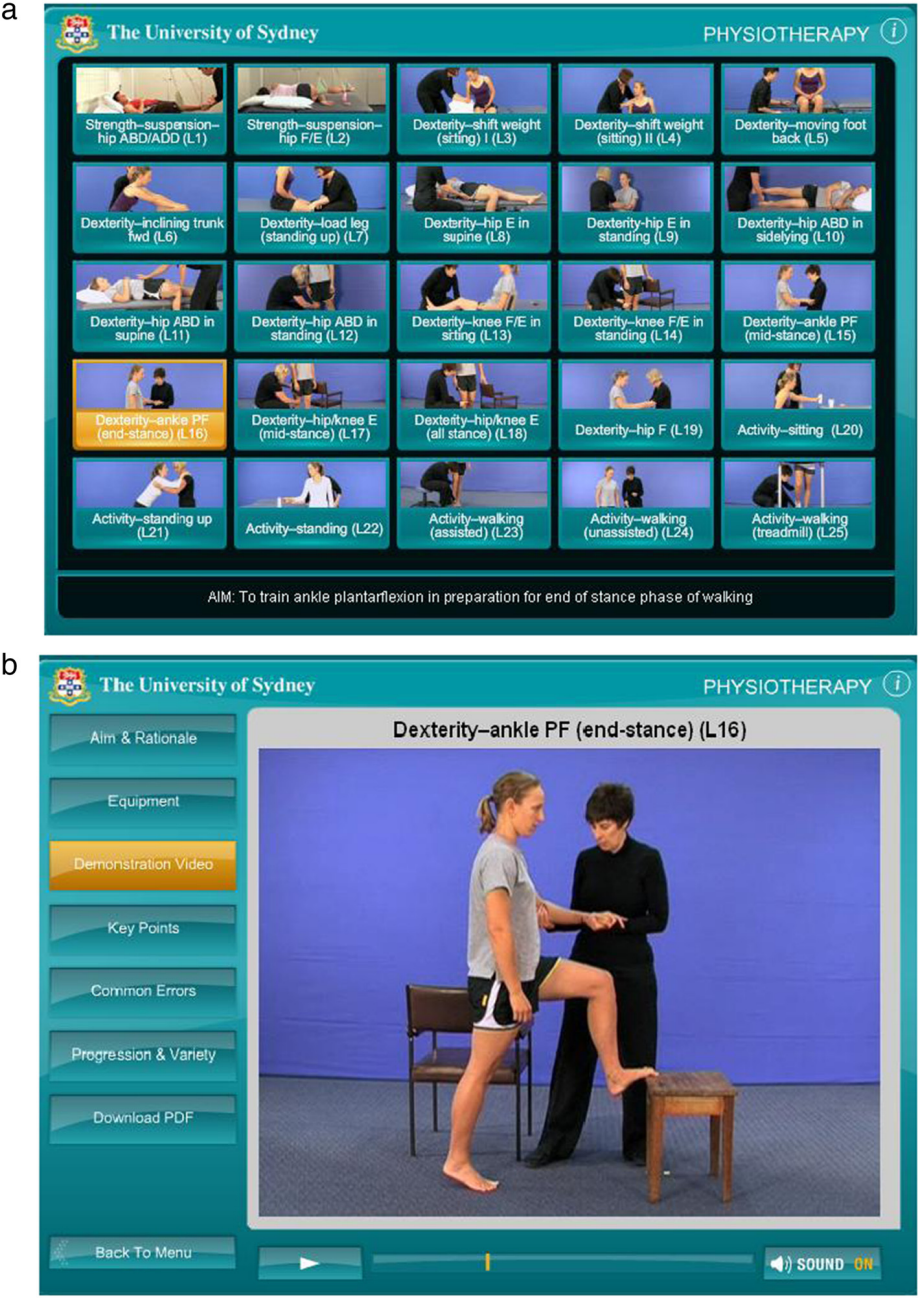
**a. The *Physiotherapy eSkills Training Online *menu page where students can select the specific practical skill.b**. The webpage for an individual practical skill from The *Physiotherapy eSkills Training Online* resource, highlighting the demonstration video, and including menu buttons for the Aim and Rationale, Equipment, Key Points, Common Errors, Progression and Variety, and a Downloadable PDF.

In order to enhance students’ learning of practical skills, the Neurological Physiotherapy Teaching Team at The University of Sydney developed the *Physiotherapy eSkills Training Online* resource to provide students with an opportunity for accurate practice outside the classroom. The resource is presented in Figure 1.

From qualitative studies in health education, we know that e-learning is well accepted [5], improves confidence in skills [6], improves self-rated performance of skills [6] and is often preferred over other modes of education [7]. However, evidence for the effectiveness of e-learning in improving the actual performance of practical skills is limited to the disciplines of nursing, midwifery, and dentistry [8,9]. Therefore, this study aimed to determine the effectiveness of the *Physiotherapy eSkills Training Online* resource. The specific research questions were:

1. Does the addition of the *Physiotherapy eSkills Training Online* resource to usual teaching improve performance of practical skills?

2. Do the students perceive the *Physiotherapy eSkills Training Online* resource as useful for learning practical skills?

## Method

### Design

**Figure 2:**
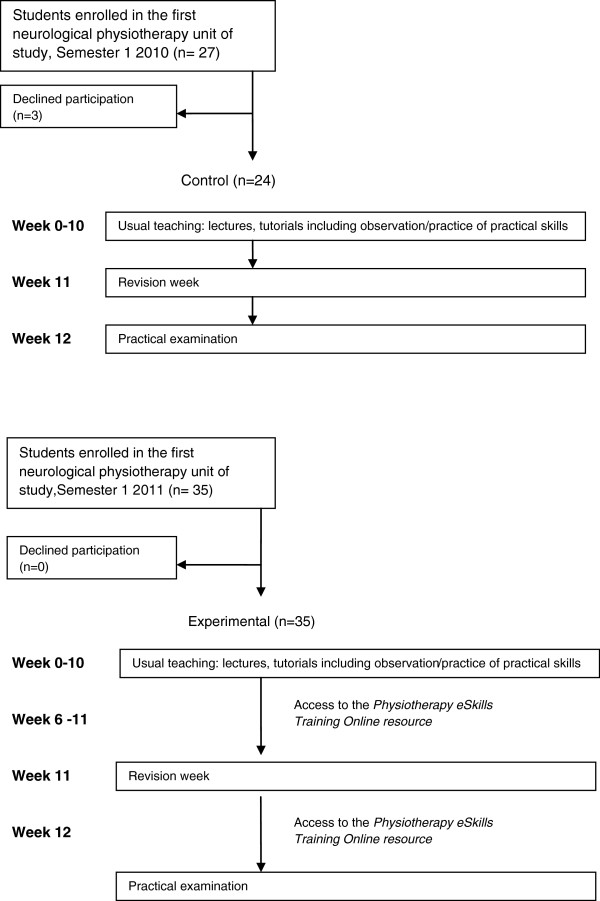
Design and flow of participants through the trial.

A non-randomised, controlled trial was carried out (Figure 2). Participants were recruited from students enrolled in a 2-year, 4-semester graduate-entry physiotherapy program (not the university where the resource was developed). Students enrolled in their first neurological physiotherapy unit of study (Semester 2, Year 1 of the program) in 2010 were recruited to the control group and those enrolled in 2011 were recruited to the experimental group. This unit of study primarily addresses the management of impairments and activity limitations associated with stroke. The experimental group therefore received access to 50 practical skills for management of stroke on the *Physiotherapy eSkills Training Online* resource over the last 5-week period of a 10-week teaching semester as well as during revision week, in addition to usual teaching. The resource incorporates 88 practical skills related to neurological physiotherapy, including practical skills related to the management of stroke, cerebellar ataxia, Parkinson’s disease, spinal cord injury and traumatic brain injury. For each practical skill, the resource includes: (i) an on demand web-streamed high quality video-clip of therapist-patient simulation; (ii) text describing the aim, rationale, equipment, key points, common errors and methods of progression; and (iii) a downloadable PDF document incorporating the online text information and a still image of the video-clip. The control group received usual teaching only. The primary outcome was the performance of practical skills after revision week at the end of the semester.

### Participants

All students enrolled in their first neurological physiotherapy unit of study in 2010 and 2011 were invited to participate. The study was approved by the University ethics committee. Participants provided informed consent prior to data collection.

### Intervention

The experimental group received the *Physiotherapy eSkills Training Online* resource as well as usual teaching. Usual teaching for the first neurological physiotherapy unit of study encompassed 2 hours of lectures in a large group, and 4 hours of tutorials in small groups (≤ 16 students) per week over 10 weeks. Tutorials incorporated explicit teaching, case-based learning, problem-based learning and live practical skill demonstration and student practice. All learning was supported by specific feedback from the tutor in relation to the learning outcomes for the class. Students also had access to pictures of practical skills in the required text and student manual. The experimental group also received access to the *Physiotherapy eSkills Training Online* resource for the last 5 weeks as well as revision week to enable revision and consolidation of practical skills at any time. The control group received usual teaching only.

### Outcome measures

The primary outcome was the performance of practical skills at the end of the semester. Performance was measured during a practical examination where the student was provided with a clinical problem requiring the demonstration of a practical skill. The student then acted as the ‘therapist’, while a fellow student simulated a ‘patient’. A standardised 25-point marking schema was used covering: informed consent (out of 1), hygiene (out of 1) rationale for the practical skill (out of 4), explanation of the practical skill (out of 2), effectiveness of the practical skill (out of 4), specificity of instructions and feedback (out of 4), progression of the practical skill (out of 4), evaluation of the effectiveness of the practical skill (out of 2) and safety (out of 3). Performance was measured by two academics with training and prior experience in the use of the marking schema.

The secondary outcome was usefulness of the *Physiotherapy eSkills Training Online* resource for learning practical skills. The students’ perceptions were measured by rating four statements on a 10-cm visual analogue scale where 0 was defined as ‘strongly disagree’ and 10 was defined as ‘strongly agree’. The statements were:

1. The *Physiotherapy eSkills Training Online* resource improved my practical skills;

2. The *Physiotherapy eSkills Training Online* resource helped me in my exam preparation;

3. The *Physiotherapy eSkills Training Online* resource has helped me or will help me on my clinical practicum;

4. I would use the *Physiotherapy eSkills Training Online* resource as a new graduate.

Participants were also invited to provide comments about the *Physiotherapy eSkills Training Online* resource.

### Statistical analysis

Mean differences (95% CI) were determined for the total practical examination score (out of 25), for each of its components and for the responses to the four statements (out of 10). Analysis by intention-to-treat was used, i.e. the data of all consenting participants in the experimental group was included in the analysis regardless of whether the *Physiotherapy eSkills Training Online* resource was utilised or not.

## Results

### Flow of participants through the trial

Fifty-nine students (95%) consented to participate in the study, 24 in the control group and 35 in the experimental group. Data were available for all participants, and all data were included in the analysis. Flow of participants through the trial is presented in Figure 2. Characteristics of the participants are presented in Table 1.

**Table 1:** Characteristics of participants

**Characteristic**	**Groups**	
	**Exp n = 35**	**Con n = 24**
Age (SD)	25 (2.3)	26 (6.4)
Gender (M:F)	13:22	8:16
English as a second language	0	2
Previous experience with content	1	0
Previous experience with online learning	35	24
Previous experience with online learning of practical skills for physiotherapy	0	0

### Compliance with trial method

Thirty-two experimental participants (91%) utilised the *Physiotherapy eSkills Training Online* resource at least once.

### Effect of *Physiotherapy eSkills Training Online* resource

Group data are presented in Table 2. The experimental group scored 1.6 points out of 25 (95% CI −0.1 to 3.3) higher than the control group in the practical examination. In addition, the experimental group scored 0.5 points out of 4 (95% CI 0 to 1.1) higher than the control group for the component ‘effectiveness of the practical skill’ and 0.6 points out of 4 (95% CI 0.1 – 1.1) higher for the component ‘rationale for the practical skill’.

**Table 2:** Mean (SD) of practical examination marks for each group and difference (95% CI) between groups

**Outcome**	**Groups**		**Difference between groups**
	**Exp n = 35**	**Con n = 24**	**Exp minus Con**
Total mark /25	19.7 (2.8)	18.1 (3.6)	1.6 (−0.1 to 3.3)
Informed Consent /1	0.9 (0.3)	0.9 (0.3)	0 (−0.2 to 0.1)
Rationale for the practical skill /4	2.7 (0.8)	2.1 (1.1)	0.6 (0.1 to 1.11)
Explanation of the practical skill /2	1.8 (0.4)	1.6 (0.7)	0.2 (−0.1 to 0.5)
Effectiveness of the practical skill /4	2.9 (0.8)	2.4 (1.2)	0.5 (0 to 1.1)
Specificity of instructions and feedback /4	2.9 (0.8)	2.8 (0.9)	0.1 (−0.4 to 0.5)
Progression of the practical skill /4	3.1 (0.6)	3.2 (0.8)	−0.1 (−0.5 to 0.3)
Evaluation of the effectiveness of the practical skill /2	1.9 (0.2)	1.8 (0.6)	0.2 (−0.1 to 0.4)
Safety /3	2.4 (0.7)	2.4 (0.9)	0.1 (−0.4 to 0.5)
Hygiene /1	1.0 (0)	1.0 (0)	0 (0 to 0)

The experimental group rated the *Physiotherapy eSkills Training Online* resource for:

1. improving their practical skills as 8.4 out of 10 (95% CI 8.0 to 8.7)

2. helping in examination preparation as 8.7 out of 10 (95% CI 8.3 to 9.1)

3. helping on neurological clinical practicum as 7.6 out of 10 (95% CI 7.0 to 8.2)

4. usefulness as a new graduate as 6.6 out of 10 (95% CI 5.8 to 7.4)

Students provided the following statements about the value of the resource:

"“[The resource] was VERY handy and useful”"

"“[The] format [was] great for learning – delicious!”"

Students provided the following statements regarding incorporation of video simulations into the *Physiotherapy eSkills Training Online* resource:

"“It was highly beneficial having a video to remind us of the skills we learnt in class”"

"“[It was] great to have a visual tool”."

## Discussion

This study examined the effectiveness of the addition of an online resource to usual teaching in improving performance of practical skills in physiotherapy students. There was a trend towards students who had access to the *Physiotherapy eSkills Training Online* resource scoring higher in the practical examination than students who had usual teaching only. The higher marks were accounted for mostly by the components of ‘rationale for the practical skill’ and ‘effectiveness of the practical skill’. Furthermore, the students perceived that the online resource was very useful for learning practical skills.

The *Physiotherapy eSkills Training Online* resource was delivered in addition to usual teaching which consisted of high face-to-face teaching hours, supported by demonstration of, practice of, and specific feedback about practical skills. This usual teaching is already effective given that the average practical examination mark before implementation of the online resource was 18 out of 25. In light of this, the trend towards a 1.6 mark increase represents a 23% increase out of the 7 remaining marks. This improvement is probably the result of the resource being online since it enabled the opportunity to view, revise and consolidate the exact practical skills required, in the physical and social context of the students’ choice. These results raise the question of whether the *Physiotherapy eSkills Training Online* resource could be used to decrease the labour intensive tutorial system of teaching physiotherapy students the practical skills required for managing patients with neurological disorders. Future research to examine whether face-to-face teaching can be streamlined by utilising this online resource as a replacement for some of the small group teaching is warranted.

The improvement observed in the total practical examination mark is accounted for largely by an increase in the components ‘effectiveness of the practical skill’ and ‘rationale for the practical skill’. There are no similar findings reported in the literature about e-learning. There could be several reasons that students' ability to effectively perform the practical skill was enhanced by the *Physiotherapy eSkills Training Online* resource, the most important of which is likely to be the observation of therapist-patient simulation via online video-clip streaming. Video modelling has been demonstrated to improve performance of skills ranging from gymnastics in healthy individuals to smiling in people who have facial paralysis [10,11]. Moreover, patient simulation has been demonstrated to increase knowledge compared with text-only electronic formats in health education [12]. It is encouraging that the students’ ability to provide a rationale for the practical skill was also improved. This may be as a result of the *Physiotherapy eSkills Training Online* resource containing supporting text describing the aim, rationale, equipment, key points, common errors and methods of progression; and a downloadable PDF document incorporating the online text information as well as the video-clip of the therapist-patient simulation. Importantly, the resource relates each practical skill to the specific impairments and activity limitations of neurological conditions in a clinical context. There is evidence that e-learning incorporating clinical scenarios improves knowledge acquisition and retention [13]. There was no increase in marks in the components ‘specificity of instructions and feedback’ or ‘progression of the practical skill’ despite the inclusion of these aspects in the *Physiotherapy eSkills Training Online* resource. However, prior to the implementation of the online resource, the average mark for feedback was 2.8 out of 4 and for progression was 3.2 out of 4, leaving less room for improvement than other components.

The *Physiotherapy eSkills Training Online* resource was well utilised with 91% of students in the experimental group using the online resource for learning practical skills. There was strong agreement that the resource contributed to improvements in practical skills, and assisted in exam preparation. Students also agreed that the resource would be useful for clinical practice both as a student and as a new graduate.

These findings are consistent with previous findings that online educational delivery can result in positive learning experiences for students [6,9,14] and supports the suggestion that self-controlled video modelling enhances learning [4].

There are several limitations to this study. Firstly, it was a non-randomised trial because it was ethically and logistically difficult to restrict access to the Physiotherapy eSkills Training Online resource for some students and not others in the same cohort, so the control and experimental blocks occurred at different times. However, the blocks were run in consecutive years, and no changes to the usual teaching occurred. Secondly, there was no blinding of participants, teachers or assessors. To minimise the consequences of lack of assessor blinding, the same standardised marking schema and assessors were used for the practical examination in each block. Thirdly, the number of participants in the study was small, leading to a lack of statistical power. A fully powered study would need a total of 162 participants to detect a mean difference of 1.6 marks, with a standard deviation of 3.6, at a significance level of 0.05. Fourthly, the frequency with which the experimental group accessed the *Physiotherapy eSkills Training Online* resource was not quantified. Finally, the students were assessed within 2 weeks of the end of teaching, so the question of whether the online resource was also effective in improving performance of practical skills during clinical practice remains. These factors suggest that the results should be interpreted with caution.

## Conclusion

Access to an online resource in addition to usual teaching appeared to be effective in improving performance of practical skills in physiotherapy students. This improvement is largely accounted for by an improvement in providing a rationale for the practical skill, as well as in the effective performance of the skill, reflecting both clinical reasoning and skill performance. The resource was considered very useful by physiotherapy students. This suggests that the development of online video simulations based on clinical scenarios may be useful in teaching and learning practical skills.

## Competing interests

LA, CD and CC were involved in the development of the *Physiotherapy eSkills Training Online* resource. The remaining authors declare that they have no competing interests.

## Authors’ contributions

EP, LA and RS participated in conception and design of the trial. EP, LA and RS are the main investigators. EP, LA and RS drafted the manuscript. LA performed all statistics. All authors critically revised the manuscript and read and approved the final manuscript.

## Pre-publication history

The pre-publication history for this paper can be accessed here:

http://www.biomedcentral.com/1472-6920/12/119/prepub
